# Targeting CD47 as a Novel Immunotherapy for Breast Cancer

**DOI:** 10.3389/fonc.2022.924740

**Published:** 2022-07-04

**Authors:** Can Chen, Runlu Wang, Xi Chen, Yulong Hou, Jingting Jiang

**Affiliations:** ^1^ Department of Oncology, The Third Affiliated Hospital of Soochow University, Changzhou, China; ^2^ Respiratory Division, The Second Hospital of Hebei Medical University, Shijiazhuang, China; ^3^ Department of Dermatology, First People’s Hospital, Huzhou, China; ^4^ Department of Surgery, Huzhou Central Hospital, Huzhou, China; ^5^ Department of Tumor Biological Treatment, The Third Affiliated Hospital of Soochow University, Changzhou, China

**Keywords:** CD47, SIRPα, breast cancer, immunotherapy, immune checkpoint inhibitors

## Abstract

Nowadays, breast cancer has become the most common cancer worldwide with a high mortality rate. Immune checkpoint blockade holds great promise in tumor‐targeted therapy, and CD47 blockade as one immune therapy is undergoing various preclinical studies and clinical trials to demonstrate its safety and efficacy in breast cancer. In this review, we summarized different therapeutic mechanisms targeting CD47 and its prognostic role and therapeutic value in breast cancer.

## Introduction

Breast cancer has become the most common cancer worldwide, with nearly 2.3 million new cases in 2020 (1). Despite significant advances in diagnostic techniques and treatment modalities, breast cancer mortality remains high with more than 600 000 patients dying each year (2). Therefore, novel and more effective therapies are still urgently in need.

Since the functional change of immune system plays an important role in the occurrence and progression of breast cancer, immunotherapy especially the blockade of immune checkpoints leads to new breakthroughs (3–5). The development of immune checkpoint inhibitors (ICIs) targeting the adaptive immune system, such as programmed cell death protein-1 (PD-1) and its ligand PD-L1, and cytotoxic T-lymphocyte-associated antigen 4 (CTLA-4), has improved outcomes in patients with advanced metastatic breast cancer and triple-negative breast cancer (TNBC) (6, 7). Although ICIs monotherapy can enhance T cell-mediated immunity, the overall response rate (ORR) is generally less than 30% (7–11). The inhibition of immune checkpoints targeting the innate immune system offers a new solution. Increasing evidence indicates that CD47 acts as a dominant “don’t eat me” signal, enabling tumor cells to escape from macrophage-mediated phagocytosis (12–14). Currently, CD47 is an attractive target for the development of new anti-cancer therapeutics, including options against breast cancer.

## Structure and Biological Function of CD47

CD47, originally found to be expressed on red blood cells (RBCs), is a 50 kDa transmembrane protein known as integrin-associated protein (IAP) (15). Structurally, CD47 consists of an extracellular N-terminal IgV domain, five highly hydrophobic transmembrane segments, and a short cytoplasmic tail (16).

**Figure 1:**
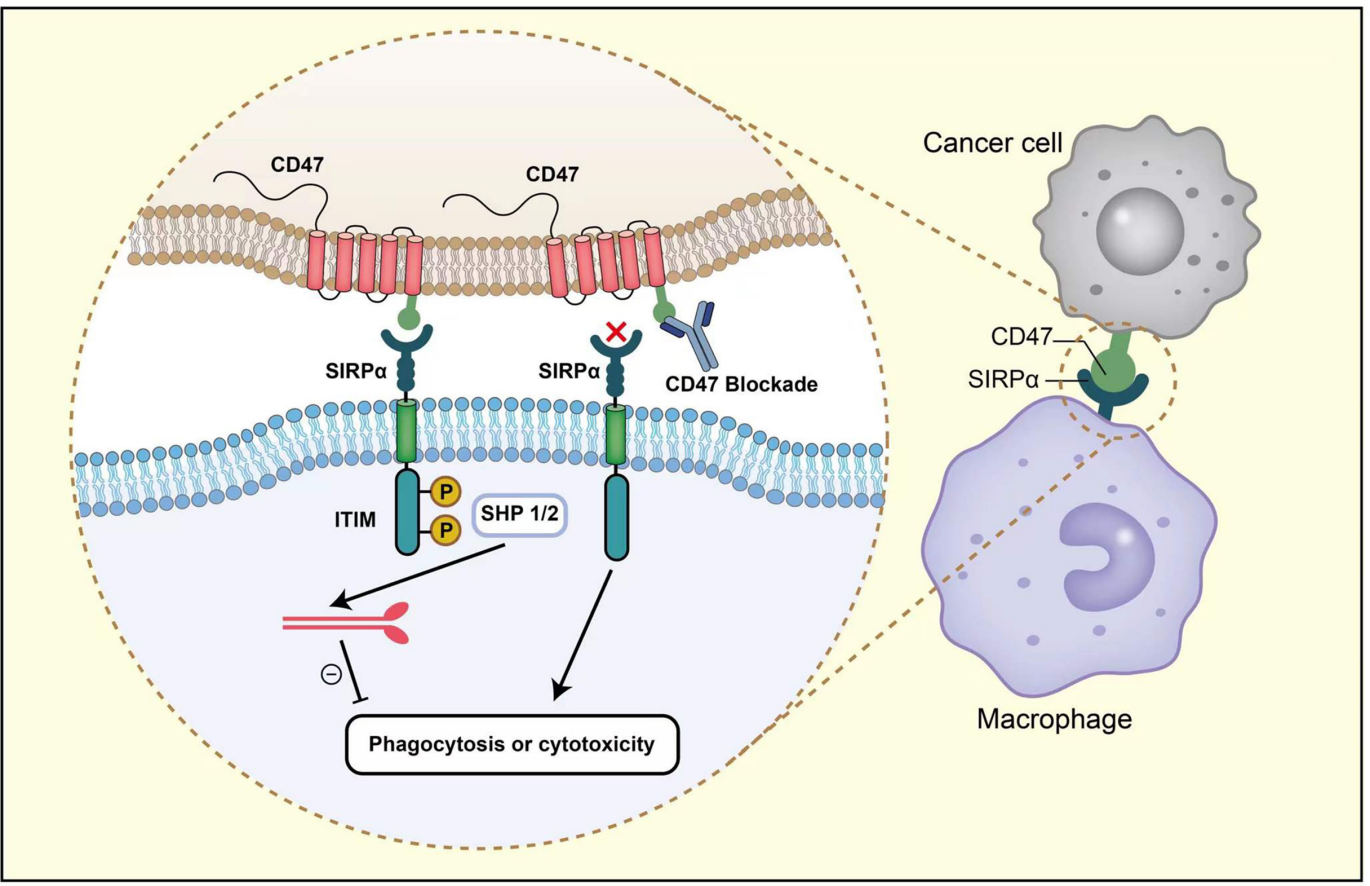
After the interaction between CD47 and SIRPα, two tyrosine residues from ITIM in the intracellular domain of SIRPα become phosphorylated. The phosphorylation activates SHP1 and SHP2, leading to the dephosphorylation of myosin IIA, thereby preventing macrophage phagocytosis. Anti-CD47 antibodies could block CD47-SIRPα axis and promote phagocytosis.

It was not until 1999 that CD47 was identified as a ligand of signal regulatory protein-alpha (SIRPα) expressed on myeloid cells, including macrophages (17). The extracellular IgV domain of CD47 binds to SIRPα and initiates the phosphorylation of two tyrosine residues from immunoreceptor tyrosine-based inhibitory motif (ITIM) in the intracellular domain of SIRPα (18) (19–21). The phosphorylation of ITIM subsequently recruits and activates phosphatases SHP1 and SHP2 (22–24). This signaling cascade results in the dephosphorylation of myosin IIA, thereby inhibiting cytoskeleton rearrangement, which is a necessary step for macrophage phagocytosis of target cells (25) (**Figure 1**).

The role of CD47 in immune recognition and phagocytosis was first described by Oldenborg et al. that red blood cells (RBCs) from CD47^-/-^ mice were rapidly cleared when infused into wild-type recipient mice, and this effect was reversed when macrophages were depleted with clodronate liposome (26). Another study found that when RBCs senesce, CD47 expression decreased, and senescent erythrocytes lacking CD47 were considered ‘foreign’ and were rapidly cleared by macrophages in the spleen (27). These results showed that erythrocyte survival was highly dependent on CD47.

Accumulating data suggest that the CD47-SIRPα axis plays an important role in suppressing tumor phagocytosis by regulating the innate immune response. Knauf S et al. first identified the expression of CD47 on ovarian tumors as early as 1986 (28), and a series of studies subsequently confirmed that CD47 was highly expressed in both hematological and solid malignancies, including non-Hodgkin’s lymphoma (NHL) (29), chronic myeloid leukemia (CML) (17), myeloma (30), osteosarcoma (31), breast cancer (32), and other solid tumors. Overexpressed CD47 interacts with SIRPα on myeloid cells to help multiple malignant tumors escape immunosurveillance (33). The disruption of CD47-SIRPα axis leads to the failure of SIRPα phosphorylation, thereby promoting phagocytosis by macrophages. During this process, antigen-presenting cells (APCs) also initiate cross-priming, activating the adaptive immune system (34). Numerous studies have shown that anti-CD47 antibody significantly enhanced the function of macrophage phagocytosis (35, 36), dendritic cell (DC) antigen presentation (37, 38), and NK cell-mediated killing (39). Overall, CD47-SIRPα axis may mediate the link between innate and adaptive immunity.

## Regulation of CD47 Expression in Breast Cancer

At the transcriptional level, more understanding about the regulation of CD47 expression in breast cancer has been described. The stimulation of tumor necrosis factor (TNF) inflammatory pathway activates nuclear factor-κB (NF-κB), which directly binds to a super enhancer (SE) site near the CD47 gene, promoting CD47 gene transcription. Contrarily, the blockade of TNF-α signaling has been shown to reduce CD47 expression and induce macrophage phagocytosis (40). Hypoxia-inducible factor 1 (HIF-1) binds to the CD47 promoter, activating gene transcription and increasing CD47 expression in breast cancer cells. Moreover, when cocultured with HIF-1-deficient breast cancer cells, the phagocytosis of macrophages was significantly enhanced (41). Using human and mouse models of leukemia and lymphoma, Casey et al. observed that MYC induced the transcription of both CD47 and PD-L1 (42). Notably, the regulatory effect of MYC on CD47 in breast cancer requires further studies. In conclusion, more understanding of CD47 expression regulation is very meaningful for optimizing CD47-related tumor targeted therapeutics.

## CD47 Expression in Breast Cancer and its Correlation With Clinical Outcome

Although CD47 expression is shown to be associated with the development of numerous tumors (43–45), its role in breast cancer is less-well characterized. Next, we summarized the currently available data in breast cancer.

In 2010, Nagahara et al. first reported CD47 as a prognosis biomarker of breast cancer; compared with controls, breast cancer patients have higher CD47 mRNA, and the high CD47 expression in bone marrow were correlated with poor survival. They believed that determining CD47 expression levels in bone marrow or peripheral blood contributed to predict the number of circulating tumor cells that escaped from the immune system, which is indicative of the presence of micrometastases (46). Yuan et al. evaluated CD47 expression using immunohistochemistry and observed that CD47 expression in breast cancer samples was significantly associated with advanced tumor node metastasis (TNM) stage, histological grade, estrogen receptor (ER) status, progesterone receptor (PR) status, and recurrence. However, high CD47 expression had a limited correlation with reduced 5-year disease-free survival (47). An analysis of 353 breast cancer patients and a public data set showed that the high CD47 mRNA levels were correlated with poor-prognosis molecular subtypes (basal, Her2/Neu^+^) and adverse clinicopathological parameters (high-grade, ER^-^, PR^-^). Moreover, in Her2/neu^+^ breast cancer patients treated with trastuzumab plus vinorelbine, the expression level of CD47 was negatively correlated with the pathological response to treatment, and CD47 was significantly reduced in the complete responders (48). By analyzing two independent datasets of 1954 breast cancer patients, Zhang et al. demonstrated that an increase in CD47 mRNA was associated with a significant decrease in overall survival (OS). The authors also reported that HIF-1 raised CD47 expression to promote breast cancer cells escape from macrophage phagocytosis (41). In a study by Baccelli et al., there was a 7.4-year difference in mean OS between CD47 positive and negative patients. Moreover, CD47 was strongly associated with lymph node metastasis (49).

In triple-negative breast cancer, CD47 expression showed 2.3-fold higher in cancer stem cells (CSCs) than the normal counterparts by Gene Set Enrichment Analysis, and this upregulation was closely related to tumor growth (50). One study revealed that CSCs increased CD47 expression to avoid immune-mediated elimination during conventional anti-tumor therapy (51). When CD47 declined, CSCs were significantly reduced in a dose-dependent manner (41, 52). Yuan et al. examined CD47 expression in 97 breast cancer tissues, and they reported that the positive rate of CD47 in TNBC tissue was significantly higher than that in benign breast lesions, and CD47 overexpression positively correlated with TNBC metastasis and recurrence (53). Many other studies have also shown that CD47 was highly expressed in breast tumors, especially in TNBCs (54). Baccelli et al. demonstrated that overexpressed biomarkers including CD47, EpCAM, CD44, and MET in breast CSCs were strongly associated with decreased OS and increased number of metastatic sites in metastatic breast cancer (55).

## Mechanism of Action and Implications of Targeting CD47-SIRPα Axis in the Breast Cancer Microenvironment

**Figure 2:**
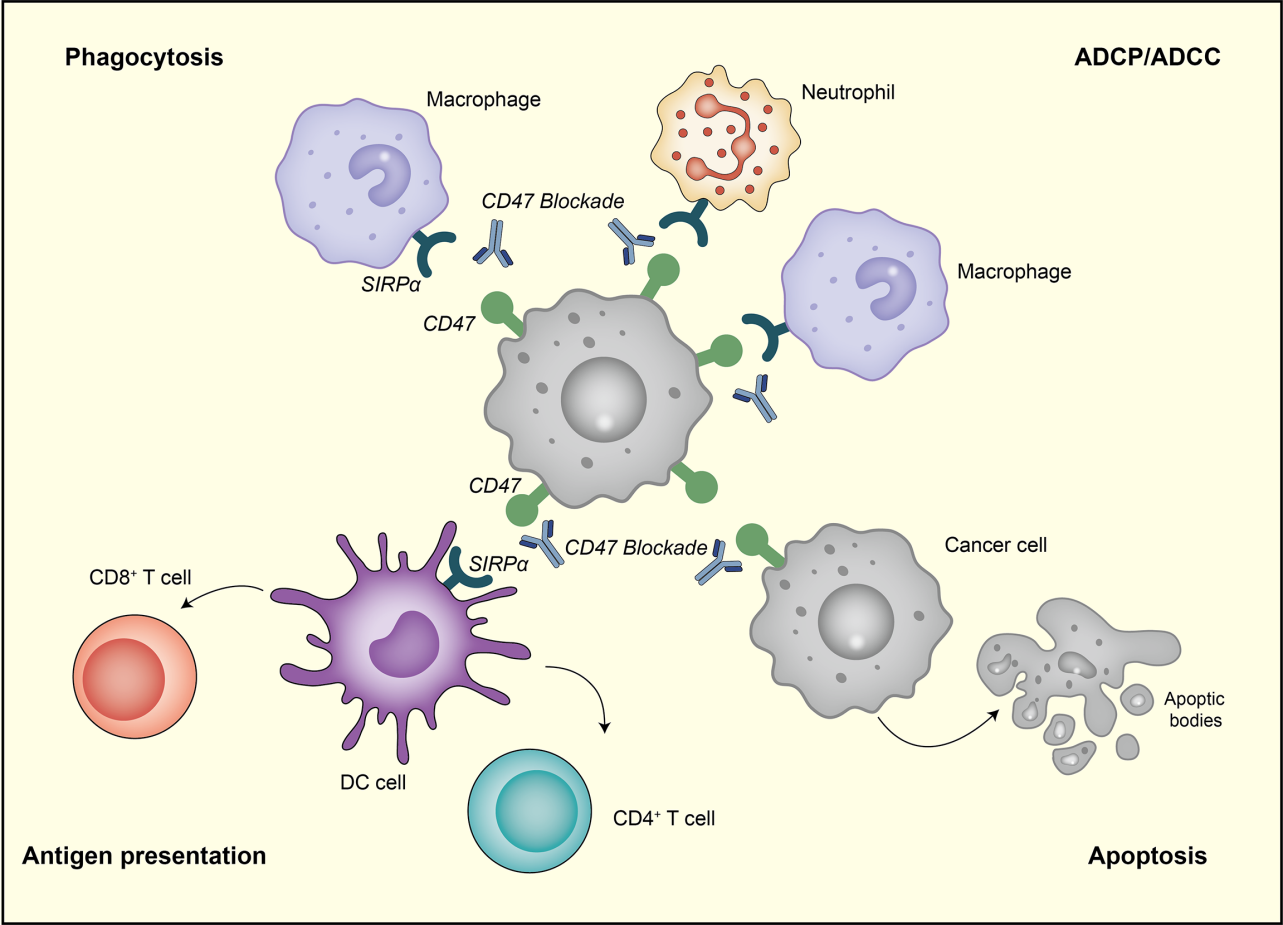
The therapeutic targeting of CD47-SIRPα pathway can cause the elimination of breast cancer cells through the following four pathways. First, the inhibition of CD47-SIRPα could enhance tumor cell phagocytosis by macrophage. Second, anti-CD47 antibody enables the phagocytic uptake of tumor cells by dendritic cells and subsequent antigen presentation to CD4 ^+^ and CD8 ^+^ T cells, thereby stimulating anti-tumor adaptive immune response. Third, anti-CD47 antibody eliminates tumor cells *via* traditional Fc-dependent mechanisms, including ADCC and ADCP. Fourth, anti-CD47 antibody stimulates tumor cell apoptosis through a caspase-independent mechanism.

The occurrence and metastasis of tumors are closely related to the internal and external environment of tumor cells, which refers to tumor microenvironment. Tumor cells can maintain the survival condition through autocrine and paracrine. Additionally, by changing the microenvironment through immunity, the body can restrict and affect tumor development. Tumor microenvironment is now recognized as a potential therapeutic target. The inhibition of CD47-SIRPα axis in the tumor microenvironment facilitates the elimination of cancer cells mainly through the following four pathways (**Figure 2**).

In the first pathway, anti-CD47 antibody disrupts anti−engulfment signal, promoting M1/M2 macrophages-mediated phagocytosis and shifting the immunosuppressive phenotype of tumor-associated macrophages (TAMs) toward M1 subtype *in vivo (* 32, 56). Feliz-Mosquea et al. reported that CD47 blockade significantly increased macrophage infiltration and phagocytosis on breast cancer cells (57). Zhang et al. came to a similar conclusion that the knockdown of CD47 expression increased macrophage-mediated cytotoxicity toward breast cancer cells, and the level of CD47 was negatively correlated with the degree of phagocytosis (41).

In the second pathway, the inhibition of CD47-SIRPα axis enhances the antigen presentation ability of DC and antigen is subsequently presented to CD4^+^ and CD8^+^ T cells, leading to the activation of adaptive immune response (58, 59). Recently, Kosaka et al. suggested that the combination treatment of cGAMP and anti-CD47 mAb induced effective anti-tumor immune responses through the activation of monocyte/macrophage phagocytosis and adaptive immune response, which relied on STING and type I IFN signaling. This combination therapy also leads to immune memory and systemic anti-tumor immune responses (60).

In the third pathway, anti-CD47 antibody could eliminate breast cancer cells *via* traditional Fc-dependent mechanisms, including neutrophil-mediated antibody-dependent cellular cytotoxicity (ADCC) and macrophage-mediated antibody-dependent cellular phagocytosis (ADCP) (12, 61). A study by Matlung et al. demonstrated that targeting CD47-SIRPα could further improve ADCC by a cytotoxicity mechanism identified as trogoptosis (62). Zhao et al. proved that B6H12, a murine antibody against human CD47, can enhance ADCC activity (48). In preclinical models of HER2^+^ breast cancer, CD47 blockade significantly increased ADCP and enhanced trastuzumab therapeutic outcomes (63).

In the fourth pathway, the inhibition of CD47 or SIRPα can induce tumor cell apoptosis (64, 65), which could be attributed to the direct ligation of CD47 rather than the caspase-dependent pathway (66, 67). In breast cancer, anti-CD47 antibody mediates direct apoptosis of tumor cells, involving the regulation of cAMP levels *via* heterotrimeric Gi with subsequent effects mediated by PKA. Notably, this effect can be effectively blocked by any drug that maintains intracellular cAMP levels and PKA activity (68).

## Targeting CD47 in Breast Cancer Treatments

While SIRPα-CD47 signaling cascade remains incompletely understood, the value of targeting CD47 in tumor treatment has been increasingly confirmed, and the clinical studies on CD47 mAbs have made rapid progress (**Table 1**). Here, we reviewed and summarized recent advances in CD47 antibodies in breast cancer treatment (**Table 2**).

**Table 1:** Clinical trials targeting CD47 registered with the National Clinical Trials Registry (NCT) system.

Drug	CD47 Isotype	Mechanism	Malignancy type	Phase	enrollment	Clinical trial ID
AK117	IgG4	Anti-human CD47 mAb	Neoplasms Malignant MDS AML Neoplasms Malignant Advanced Malignant Tumors Advanced Malignant Tumors TNBC	1 1/2 1/2 1 1/2 1/2 2	162 190 160 159 114 130 80	NCT04728334 NCT04900350 NCT04980885 NCT04349969 NCT05229497 NCT05235542 NCT05227664
ALX148	IgG1	Anti-human CD47 mAb	Microsatellite Stable Metastatic Colorectal Cancer B-cell NHL Higher Risk MDS Advanced Solid Tumors NHL Head and Neck Squamous Cell Carcinoma Head and Neck Squamous Cell Carcinoma Gastric or Gastroesophageal Junction Adenocarcinoma	2 1/2 1/2 1 2 2 2/3	80 52 173 174 168 183 450	NCT05167409 NCT05025800 NCT04417517 NCT03013218 NCT04675333 NCT04675294 NCT05002127
AO-176	IgG2	Anti-human CD47 mAb	MM Solid Tumor	1/2 1/2	157 183	NCT04445701 NCT03834948
BAT7104	IgG-like	Anti-CD47/PD-L1 bifunctional antibody	Advanced Solid Tumors	1	29	NCT05200013
CC-90002	IgG4	Anti-human CD47 mAb	AML MDS Hematologic Neoplasms	1 1	28 60	NCT02641002 NCT02367196
CPO107	IgG1	Anti-CD20/CD47 bifunctional antibody	NHL	1/2	75	NCT04853329
DSP107	IgG4	Bi-functional CD47 and 41BB fusion protein	Hematological Malignancies Non-Small Cell Lung Cancer AML/MDS CML	1 2 1 2	100 36	NCT04440735 NCT04937166
Hu5F9−G4 (Magrolimab)	IgG4	Anti-human CD47 mAb	Solid Tumor AML AML AML Solid Tumor Colorectal Cancer Ovarian Cancer NHL Neuroblastoma Osteosarcoma Hematological Malignancies AML AML NHL	1 1 1 1 2 1 1/2 1 1 3 1/2 1	88 13 20 78 34 178 82 287 520 98 30	NCT02216409 NCT03922477 NCT02678338 NCT02953782 NCT03558139 NCT02953509 NCT04751383 NCT03248479 NCT04313881 NCT04435691 NCT03527147
HX009	IgG4	Anti-CD47/PD-1 bifunctional antibody	Relapsed/Refractory Lymphoma Advanced Solid Tumor Advanced Solid Tumor	1/2 1 2	99 21 210	NCT05189093 NCT04097769 NCT04886271
IBI188	IgG4	Anti-human CD47 antibody	Advanced Malignancies Advanced Malignancies MDS AML AML MDS Solid Tumors Lung Adenocarcinoma Osteosarcoma	1 1 1 1/2 1 1	49 42 12 126 58 120	NCT03717103 NCT03763149 NCT04485065 NCT04485052 NCT05263271 NCT04861948
IBI322	IgG4	Anti-CD47/PD-L1 bifunctional antibody	Advanced Malignant Tumors Lymphomas Advanced Solid Tumor Hematologic Malignancy Advanced Malignancies Myeloid Tumor Small Cell Lung Cancer Non-Small Cell Lung Cancer	1 1 1 1 1 2 2	51 36 230 218 124 40 80	NCT04338659 NCT04912466 NCT04795128 NCT04328831 NCT05148442 NCT05296603 NCT05296278
IMC-002	IgG4	Anti-human CD47 antibody	Advanced Cancer Solid Tumor Lymphoma	1 1	24 24	NCT05276310 NCT04306224
IMM0306	IgG1	Anti-CD20/CD47 bifunctional antibody	NHL	1	90	NCT04746131
PF-07257876	IgG4	Anti-CD47/PD-L1 bifunctional antibody	Non-Small Cell Lung Cancer Head and Neck Squamous Cell Carcinoma Ovarian Cancer	1	90	NCT04881045
SG2501	Unknown	Anti-CD38/CD47 bifunctional antibody	Hematological Malignancy Lymphoma	1	72	NCT05293912
SHR-1603	IgG4	Anti-human CD47 antibody	Physiological Effects of Drugs Neoplasms by Histologic Type NHL	1	128	NCT03722186
SRF231	IgG4	Anti-human CD47 antibody	Advanced Solid Cancers Hematologic Cancers	1	148	NCT03512340
STI-6643	IgG4	Anti-human CD47 antibody	Solid Tumor Relapsed Solid Neoplasm Refractory Tumor	1	24	NCT04900519
TG-1801	IgG4	Anti-CD19/CD47 bifunctional antibody	NHL NHL CLL	1 1	16 60	NCT03804996 NCT04806035
TJ011133	IgG4	Anti-human CD47 antibody	MM AML/MDS	1 1	163 120	NCT04895410 NCT04912063
TTI-621	IgG1	SIRPα-IgG1 Fc Fusion Proteins	Hematologic Malignancies Solid Tumor Leiomyosarcoma MM Solid Tumors Mycosis Fungoides	1 1/2 1 1	250 80 32 56	NCT02663518 NCT04996004 NCT05139225 NCT02890368
TTI-622	IgG4	SIRPα-IgG4 Fc Fusion Proteins	MM Epithelial Ovarian Cancer Fallopian Tube Carcinoma Primary Peritoneal Carcinomas	1 1/2	32 50	NCT05139225 NCT05261490
ZL-1201	IgG4	Anti-human CD47 antibody	Advanced Cancer	1	66	NCT04257617

MM, multiple myeloma; AML, acute myeloid leukemia; MDS, myelodysplastic syndrome; CLL, chronic lymphocytic leukemia.

**Table 2:** Targeting CD47 in breast cancer.

Treatment		Model	Reference
Anti-CD47 antibody monotherapy	Anti-CD47 Anti-CD47 Anti-CD47 Anti-CD47	Breast cancer cell lines Breast CSCs Breast cancer Breast cancer	(68) (50) (69) (32)
Anti-CD47 antibody in combination with chemotherapy	Anti-CD47+Mitoxantrone Anti-CD47+doxorubicin Anti-CD47+cabazitaxel Anti-CD47+mertansine	Breast cancer Breast cancer TNBC TNBC	(69) (57) (54) (70)
Anti-CD47 antibody in combination with tumor-targeting antibodies	Anti-CD47+trastuzumab Anti-CD47+trastuzumab Anti-CD47+trastuzumab Anti-CD47+trastuzumab Anti-CD47+sorafenib	HER2^+^ breast cancer Radioresistant HER2^+^ breast cancer ADCC-tolerant HER2^+^ breast cancer HER2^+^ breast cancer Breast cancer	(71) (72) (73) (63) (74)

### Anti-CD47 Antibody Monotherapy

In 2004, Manna et al. found that anti-CD47 mAb 1F7 could cause the death of four different breast cancer cell lines (68). Kaur et al. showed that CD47 blockade inhibited breast CSCs proliferation and asymmetric cell division (50). It is well known that CSCs play an important role in tumor survival, proliferation, metastasis, and recurrence.

Iribarren et al. demonstrated that the monotherapy of CD47 antibody could effectively reduce tumor growth and increase overall survival in AT3 breast cancer model. Regulatory T cells (Tregs) are involved in tumor development and progression by inhibiting antitumor immunity. Of note, this treatment results in a partial reduction of M2 macrophages and almost complete elimination of immunosuppressive Tregs, suggesting that CD47 blockade remodels the tumor microenvironment (69). This elimination might be attributed to CD47 expression on Tregs, and anti-CD47 antibody would increase ADCP of the targeted Tregs (75).

In orthotopic mouse breast cancer model, anti-CD47 antibody inhibited tumor growth and prevented metastasis on larger tumors, and may be curative on smaller tumors; Importantly, anti-CD47 mAbs produced no unacceptable toxicity in immune competent mice, albeit with a temporary anemia, indicating the safety of targeting CD47 (32).

### Anti-CD47 Antibody in Combination With Chemotherapy

It is important to point out that targeting CD47 can immensely enhance the anti-tumor effect of other therapeutic strategies.

Calreticulin (CRT) is the dominant pro-phagocytic signal on multiple human cancers, which facilitates cell clearance by engaging its counter receptor LDL-receptor-related protein (LRP) on phagocytes. The balance between antiphagocytic signal (i.e., CD47) and pro-phagocytic signal (i.e., CRT) ultimately determines if cancer cells will be phagocyted or not (76, 77). Anthracyclines induce the rapid translocation of CRT to the cell surface, thereby increasing the immunogenicity of tumors (78). The blockade of CD47 in combination with anthracyclines results in the activation of immunogenic cell death pathway and enhances tumor ablation *in vivo (* 57). Iribarren et al. observed that anti-CD47 antibodies and anthracycline mitoxantrone (MTX) could be favorably combined against carcinogen-induced breast cancers, and this synergistic effect inhibited tumor growth more significantly than either treatment alone (69). Feliz-Mosquea et al. used a 2-dimensional high-throughput cell proliferation assay in mouse 4T1 breast cancer model, and they concluded that targeting CD47 could reduce breast cancer growth and metastasis by activating anti-tumor innate immune response, thereby enhancing the efficacy of doxorubicin chemotherapy in vivo. In addition, anti-CD47 antibodies prevent anthracycline-mediated cardiotoxicity and tissue toxicity (57).

Recently, Cao et al. demonstrated that the combination of CD47 blockade and cabazitaxel, an FDA-approved chemotherapeutic agent (79), produced a potent anticancer effect in TNBC preclinical models, promoting Programmed Cell Removal (PrCR) of cancer cells, and inhibiting tumor development and metastasis; while the efficacy of CD47 antibody alone in inducing PrCR was not satisfactory. More importantly, they discovered that the anti-cancer effect of cabazitaxel in TNBC was due to macrophage activation rather than drug cytotoxicity toward cancer cells. Hence, the combination of CD47 blockade and cabazitaxel may be an effective strategy for TNBC treatment (54).

Numerous studies found that CD47 expression was upregulated in chemotherapy-treated TNBC cells (80, 81). Si et al. developed an innovative antibody-drug conjugates (ADCs) constructed from a specific anti-CD47 mAb and the potent cytotoxic drug-mertansine for the treatment of TNBCs following the standard cytotoxic chemotherapies. Compared with free drug (single drug not conjugated to antibody), ADC showed higher tumor suppressor potency with reduced IC50, and significantly inhibited tumor growth after chemotherapy in TNBC mouse models. Moreover, the whole blood analysis indicated that the new anti-CD47 mAb had no general immune toxicity (70).

### Anti-CD47 Antibody in Combination With Tumor-Targeting Antibodies

Anti-CD47 antibody can also be used with biologics in addition to the combination with regular chemotherapy.

In the study of Weiskopf K et al., using the Her2/neu^+^ breast cancer cell line for phagocytosis determination, the combination of trastuzumab (an anti-HER2 antibody) and high-affinity recombinant SIRPα protein FD6 or CV1 resulted in the highest level of phagocytosis, which was significantly higher than the additive effect of either agent administered alone. Furthermore, CV1-monomer combined with trastuzumab completely eliminated tumors in breast cancer xenograft model using the humanized NOD/SCID/IL-2 receptor gamma-chain(null) (NSG) mice (71).

During radiotherapy, tumors can gradually adapt to changes in the physical and chemical environment and develop radioresistance, which is the main reason for the failure of clinical radiotherapy. Candas-Green et al. found that the aggressive behavior of radioresistant breast cancer was caused by CD47-mediated anti-phagocytosis conjugated with HER2-prompted proliferation. *In vivo* experiments, the dual inhibition of CD47 and HER2 can effectively increase the radiosensitivity of radiotherapy-resistant tumors and enhance the phagocytosis of tumor cells by macrophages (72).

Impairments in trastuzumab-mediated ADCC may lead to relative resistance to trastuzumab in advanced-stage HER2^+^ breast cancer patients (82, 83). Trastuzumab could engage Fc-γ receptors (FcγR) on macrophages and promote ADCP, which can be enhanced by anti-CD47 antibody (84). The combination of anti-CD47 antibody and trastuzumab significantly suppressed the growth of ADCC-tolerant HER2^+^ breast cancers, which could represent a potential new treatment option for HER2^+^ breast cancer patients (73).

Similarly, in a study by Tsao et al., anti-CD47 antibody significantly enhanced trastuzumab-mediated ADCP and promoted TAM expansion and activation. In addition, CD47 expression was inversely associated with the survival of HER2^+^ breast cancer patients, and the tumors in human HER2^+^ breast cancer xenografts models treated with trastuzumab plus CD47 inhibition showed complete regression (63).

The highly immunosuppressive microenvironment after surgery is critical for the recurrence and metastasis of breast cancer. Recently, Huang et al. designed an injectable Double-Layer-Gel (DLG) matrix for postsurgical treatment of breast cancer. The outer layer of DLG could release sorafenib first, which reeducates TAMs and promotes an immunogenic tumor microenvironment. The inner layer, loaded with anti-CD47 antibody, enabled the sustained release of anti-CD47 antibody. They demonstrated that in breast cancer mouse model, the DLG-based strategy efficiently prevented tumor recurrence and metastasis by locally reversing immunosuppression and synergistically blocking CD47-dependent immune escape (74).

## Biosafety Problems and Future Perspectives

Due to the ubiquitous expression in normal cells (85), anti-CD47 antibodies could cause possible off-target effects, such as anemia, thrombocytopenia, and leukopenia (76). One study suggested that Hu5F9-G4, an anti-CD47 antibody, alone or in combination with other antibodies may accidentally kill normal erythrocytes, leading to anemia (86). To alleviate this adverse effect, Advani et al., proposed to give short priming low-dose of Hu5F9-G4 in combination with rituximab to selectively eliminate the aged RBCs, thereby inducing compensatory hematopoiesis (87). The wide expression of CD47 also creates an “antigen sink”, which means that larger initiation doses and/or frequenter administrations may be required to achieve effective blockade. Thus, there is an ongoing need to exploit safer solutions to overcome toxicities, and several strategies have been developed to address these issues by selectively binding to CD47 on tumor cells, including the identification of tumor-specific CD47 epitopes and the designs of bispecific antibody.

Although single CD47-targeted agents may have significant efficacy in breast cancer, data from immunocompetent mice and breast cancer xenograft models suggest that combination therapy is still required. Presently, this synergy has been shown to be effective in preclinical models, such as anti-CD47 therapy combined with chemotherapy or immune checkpoint inhibition agents. Future advances in cancer screening and precision medicine would help define which type and stage of breast cancer is most amenable to be treated with one or more specific types of anti-CD47 therapies.

## Conclusions

In conclusion, CD47 is a novel attractive target for the treatment of breast cancer, which functions as ‘don’t eat me’ signal to assist cancer cells to escape immunosurveillance. Strategies targeting the CD47-SIRPα axis demonstrate promising results for breast cancer treatment. However, there are a series of biosafety problems with such treatments, and further clinical trials are needed to determine the clinical efficacy of these strategies.

## Author Contributions

All authors listed have made a substantial, direct, and intellectual contribution to the work and approved it for publication.

## Funding

This work was supported by the National Key R&D Program [grant number 2018YFC1313400]; the Joint Research Fund for Overseas Chinese, Hong Kong and Macao Scholars [grant number 31729001]; the National Natural Science Foundation of China [grant numbers 81972869, 81902386].

## Conflict of Interest

The authors declare that the research was conducted in the absence of any commercial or financial relationships that could be construed as a potential conflict of interest.

## Publisher’s Note

All claims expressed in this article are solely those of the authors and do not necessarily represent those of their affiliated organizations, or those of the publisher, the editors and the reviewers. Any product that may be evaluated in this article, or claim that may be made by its manufacturer, is not guaranteed or endorsed by the publisher.
